# Application of Negative Pressure Therapy on Skin Grafts after Soft-Tissue Reconstruction: A Prospective Observational Study

**DOI:** 10.3390/clinpract12030044

**Published:** 2022-06-07

**Authors:** Aeshah Mandili, Abdullah Aljubairy, Bayan Alsharif, Wala Patwa, Khlood Alotibey, Sara Basha, Ziyad Alharbi

**Affiliations:** 1 Department of Surgery, King Faisal Specialist Hospital and Research Centre, P.O. Box 40047, Jeddah 21499, Saudi Arabia; mandili.aeshah@gmail.com; 2 Intensive Care Service Department, King Fahad Armed Force Hospital, P.O. Box 9862, Jeddah 21159, Saudi Arabia; dr.abdullahaljubairy@hotmail.com; 3 General Surgery Department, Security Forces Hospital, P.O. Box 14799, Mecca 21955, Saudi Arabia; bayan.m.alsharif@gmail.com; 4 General Surgery Department, International Medical Center, P.O. Box 2172, Jeddah 21451, Saudi Arabia; walaa_patwa@hotmail.com; 5 General Pediatric Department, King Abdulaziz Medical City, P.O. Box 9515, Jeddah 21423, Saudi Arabia; khlood.o.alotibey@gmail.com; 6 General Surgery Department, Heraa General Hospital, Mecca 24227, Saudi Arabia; sara_basha94@hotmail.com; 7 Plastic Surgery and Burn Unit, Dr. Soliman Fakeeh Hospital, P.O. Box 2537, Jeddah 21461, Saudi Arabia; 8 Clinical Sciences Department, Fakeeh College for Medical Sciences, P.O. Box 2537, Jeddah 21461, Saudi Arabia

**Keywords:** split-thickness skin grafts, dressings, negative-pressure wound therapy, vacuum-assisted closure, wound healing

## Abstract

A split-thickness skin graft (STSG) is one of the main tools used in the reconstruction of skin defects. Negative-pressure wound therapy (NPWT) has been widely used as adjunct therapy for wound healing for decades. Few studies have conducted the outcomes of NPWT use as a postoperative dressing for STSGs. This study aimed to compare the outcomes of the application of NPWT versus conventional dressing on STSGs after soft-tissue reconstruction. A prospective observational study was performed at the King Abdullah Medical City. A total of 18 patients with STSGs for acute or chronic skin defects were recruited. Patients from the two groups—10 patients in the NPWT group and 8 in the No-NPWT group—were postoperatively evaluated for three weeks. Assessment included the STSG take rate, wound healing, pain, infection, hematoma formation, and the need to re-graft the same recipient area. Our data demonstrated a higher mean skin graft take rate in the second and third weeks of the No-NPWT group compared to the NPWT group, but it was not statistically significant (*p* > 0.05). No significant differences between the two groups in terms of wound healing, pain, infection, hematoma formation, and the need to re-graft (*p* > 0.05) were found. Our study showed that the conventional dressing of STSGs is not inferior to NPWT. In addition, conventional dressing was shown to be easier to use and less expensive to apply, as well as having a higher skin graft take rate and lower infection rate.

## 1. Introduction

The reconstruction of soft-tissue defects with skin grafting is an indispensable procedure in plastic and reconstructive surgery. It is often used to reconstruct defects after traumatic wounds, burns, and oncologic resection, as well as to release scar contracture. A skin graft involves transferring non-vascularized free tissues with varying thicknesses from the donor site to the remote-recipient site elsewhere in the body. To assist in the development of a new blood supply and attachment to the underlying tissues in the new location, the graft must be applied to a healthy uncontaminated bed or tissue that is vascular enough to produce a bed [1]. The classification of grafts can be done according to their host–donor relationships and their thicknesses. The most common type of clinically used graft used is the autogenous graft, wherein a graft is taken from one area of an individual’s body and applied to a different area on the same person’s body. Skin graft can be either split-thickness or full-thickness. Each type has its unique preparation technique, as well as inherent advantages and disadvantages. Carrying out split-thickness grafts may require the physician to have specific skills and/or high-priced equipment. Although they are usually less cosmetically attractive, they “take” better than full-thickness grafts. Full-thickness grafts require no special skill or high-priced equipment, and their cosmetic appearance is better than that of split-thickness grafts, despite the fact that they do not take as well as split-thickness grafts [2]. Several factors have been described that affect the survival of split-thickness skin grafts (STSGs), including recipient bed quality, production of seromas or hematomas, and nutritional conditions [3]. Conventionally, STSGs are covered with non-adherent dressings along with cotton pads with or without a tie-over bolster dressing. However, the prevention of seromas or hematomas collection under grafts is not always ensured in this process.

Negative-pressure wound therapy (NPWT) or vacuum-assisted closure (VAC) has proven to be a good alternative for a regular dressing since its introduction in the early 1990s [4]. The technique involves the use of an open-cell reticulated foam dressing applied within the wound cavity and covered with an adhesive drape to create an airtight seal. A fenestrated evacuation tube is connected to a VAC pump, applying topical controlled negative pressure to the inner surface of the wound. NPWT is presumed to work via two mechanisms of action: (1) the removal of excess interstitial fluid as its excessive accumulation increases the interstitial pressure, which may result in tissue hypoxia and necrosis and thus affect wound healing; and (2) the mechanical stress to the tissue, as tissue growth and expansion are thought to be enhanced under such stress to the wound and surrounding soft tissues [5,6]. Given these benefits, many surgeons have applied NPWT to skin grafts, and this method has also been observed in some existing studies.

Blackburn et al. [7] and Schneider et al. [8] reported improved take rates due to total graft immobilization, and better and faster engraftment and vascularization of Integra over-complicated and exposed tissue defects with the use of VAC [9]. A study was conducted in 2018 to compare the clinical outcomes of NPWT versus conventional therapy on split-thickness skin after grafting which showed its effectiveness on the management, by reducing skin graft rejection, decreasing the incidence of wound complications and improving wound outcomes [10]. A randomized controlled trial (RCT) study applied NPWT in burn patients, who reported a dramatic decrease in pain (e.g., pain associated with dressing changes) and anxiety compared to traditional dressings that require more frequent changes [11]. The authors of a meta-analysis stated that NPWT compared to standard surgical dressings significantly reduced the rate of wound infections and the development of seromas in closed surgical wounds [12] However, another study conducted in 2018 showed no significant impact on wound infection [10], and bacterial colonization increased significantly with wound VAC therapy. In addition, there was no general decrease in bacterial bioburden [13]. Furthermore, recent research has revealed a significant reduction in graft lift-off via edema, exudates, sub-graft hematoma, and shear reduction when compared to traditional dressings [14]. Regarding the literature, there have only been a few reports, mainly in the plastic surgery literature, about the use of combined therapy involving NPWT and skin grafts, as well as its impact on graft-healing outcomes. This study aimed to compare wound healing, graft taking, infection, pain, and hematoma rates in patients treated with NPWT versus conventional dressing on split-thickness skin after grafting surgery.

## 2. Design and Methods

### 2.1. Study Design

A clinical observational analytical prospective cohort study was performed from July 2018 to July 2020 at the Department of Plastic and Reconstructive Surgery, King Abdullah Medical City, Saudi Arabia.

### 2.2. Participants

Patients (≥18 years of age) who planned to have split-thickness skin grafts for acute wounds, chronic wounds, or skin defects with or without NPWT application were recruited. Exclusion criteria included skin-graft surgeries in the face, neck, and genital areas, as well as patients who had active bleeding or sepsis and those who were lost to follow-up.

### 2.3. Data Collection Tools and Methods

**Figure 1:**
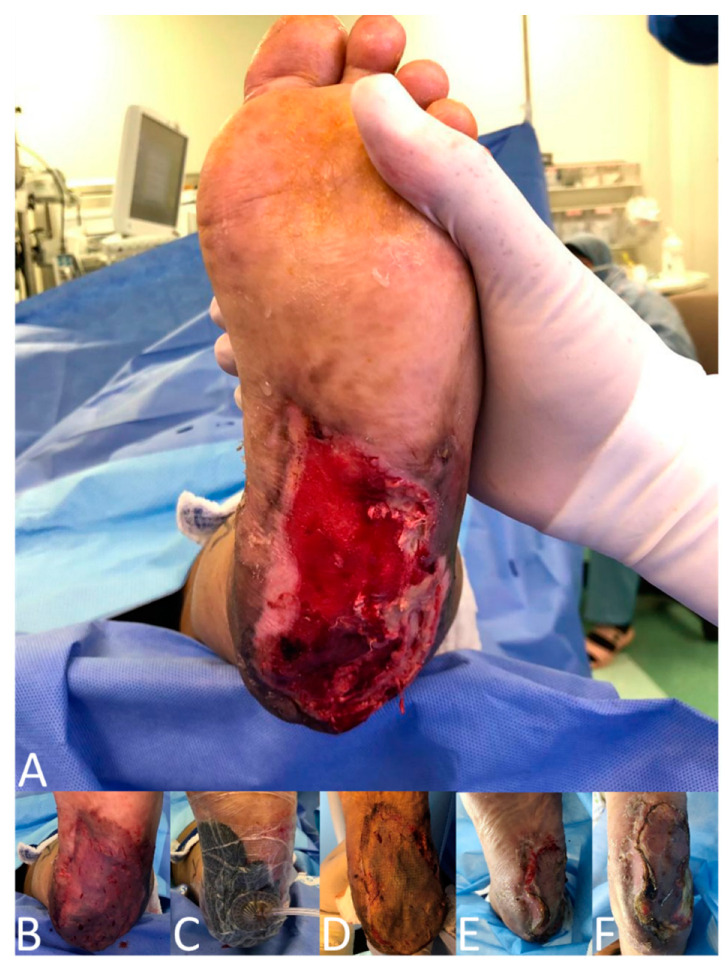
Patient in NPWT group with skin defect in the left lower extremity. (**A**) After debridement, (**B**) STSG meshed, smoothed, secured in place, (**C**) STSG covered with NPWT, (**D**) Postoperative week 1. (**E**) Postoperative week 2. (**F**) Postoperative week 3.

**Figure 2:**
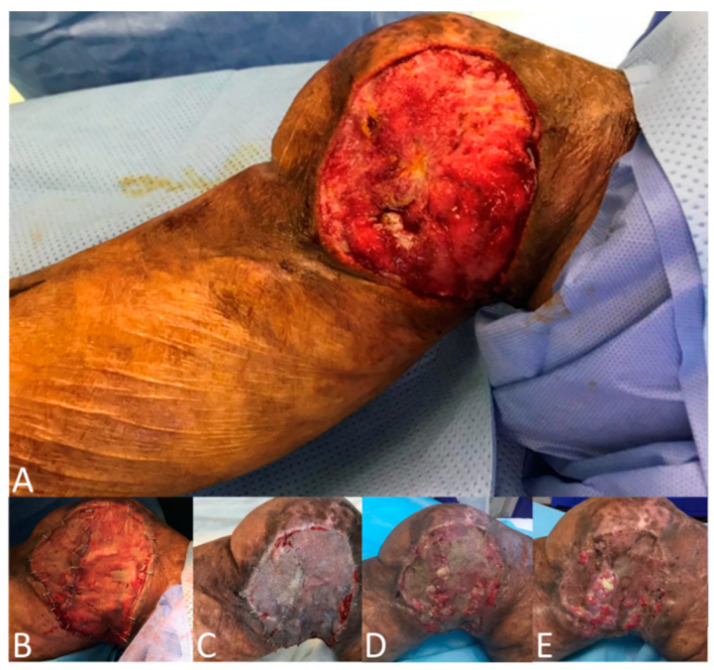
Patient in No-NPWT group with skin defect in the right lower extremity. (**A**) After debridement, (**B**) STSG meshed, smoothed, secured with staples in place, and covered with non-adherent conventional dressing, (**C**) Postoperative week 1, (**D**) Postoperative week 2, (**E**) Postoperative week 3.

To achieve the study’s objectives, a master sheet was designed by investigators, which guided the study objectives and the literature review. The data collection sheet was reviewed and revised by a research consultant and statistics expert at King Abdullah Medical City (KAMC). At baseline, for eligible patients, screening and informed consent were obtained pre-operatively. Information on patient demographics and wound characteristics was collected. Intra-operative data, including the surgeon’s choice whether to apply VAC, were recorded. The application of NPWT was based upon surgeon preference. After harvesting, meshing, and applying the autologous STSGs to the recipient sites, the STSGs were then secured to the wound beds with either absorbable sutures or staples for all participants. For all patients, the distribution of the thickness of STSGs was 0.2 mm and the distribution of MESH ratio was 1:1.5. For the No-NPWT group, non-adhesive des-infected gauze, cotton bandages, and roller gauze bandages were placed over the grafts and secured with elastic adhesive bandages or tie-over dressings as deemed necessary in a conventional dressing. Dressing changes occurred in the No-NPWT group after five days post-surgery (first dressing), with the same frequency applied every three days until 14 days then every 1 week until complete healing. For the NPWT group, as shown in Figure 1, NPWT was intraoperatively applied to the wound and maintained for five to seven days afterward. A black foam dressing was used as it is the standard sponge of NPWT and we decided to avoid silver foam which may add more anti-infection power compared to the other group. Skin graft and wound healing were assessed using the Bates–Jensen wound assessment tool (BWAT). The Present Pain Intensity (PPI) Scale was used for pain assessment. Clinical examination, blood test readings, and tissue swabs were used for infection assessment. Hematoma assessment was done via clinical examination, and the length, width, and depth of hematoma were measured. The assessments were repeated one, two, and three weeks after the skin graft surgery. High-resolution photographs of the wounds were used for documentation preoperatively, intraoperatively, and at each visit (Figure 1 and Figure 2). Following every wound assessment, patients were treated with standard wound care, including debridement, cleansing, and wound dressings during the study.

### 2.4. Approvals

This study was approved by the Institutional Review Board (IRB) of King Abdullah Medical City. A written informed consent form (ICF) that had received approval by the IRB was completed and submitted by all participants for the publication of this study. A copy of the signed ICF was obtained for each participant, and these documents are available for review by the Editor-in-Chief of this journal.

### 2.5. Sample Size and Statistical Analysis

A sample size of 28 participants was estimated using the RaoSoft^®^ online calculator (RaoSoft Inc., Seattle, WA, USA), yielding a power of 90% at a significance level of 0.05. Comparison between groups was performed via *t*-tests, and the Mann–Whitney test was used to assess the data distribution of the continuous variables. A chi-squared test used for all categorical variables. A *p*-value of less than 0.05 was considered statistically significant. All statistical analyses were carried out using SPSS statistical software, version 26 (SPSS, Chicago, IL, USA).

## 3. Results

A total of 34 patients were screened; 18 participants were enrolled in the study, 16 were excluded. Ten (55.6%) patients were in the NPWT group, and eight (44.4%) in the No-NPWT group. The demographic and wound characteristics of our participants were compared between the NPWT and No-NPWT groups, as presented in Table 1. By gender, more than half of the cases were males (12, 66.7%) and six (33.3%) were females. Half of the cases (50%) were residents in Makkah, and the majority (88.8%) were Saudi. Four patients (40.0%) in the NPWT group and one (5.5%) in the No-NPWT group suffered from heart disease (HD). Half of the participants (nine, 50%) had acute wounds, and the other half had chronic wounds. Evaluations of the wound sites in the study participants showed that most of the wounds (10, 55.6%) were in the lower limbs, followed by the upper limbs (6, 33.4%), thorax (1, 5.5%), and abdomen (1, 5.5%). The differences between the wound sites in the NPWT group versus the No-NPWT group were significant (*p* = 0.04). However, there were no striking significant statistical differences between the two groups in terms of patients’ demographics, co-morbidities, wound types, preoperative wound surface areas, and BWAT scores (Table 1).

**Figure 3:**
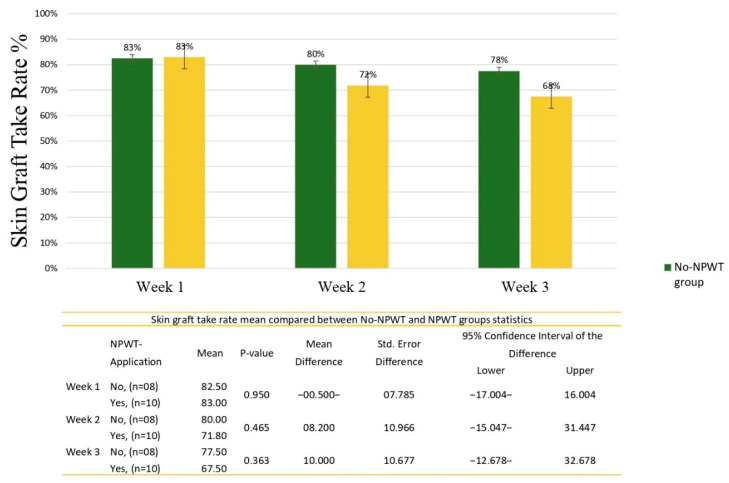
Skin-graft take rate mean compared between the No-NPWT and NPWT groups.

The evaluation of skin-graft take rates revealed a mean take rate of the No-NPWT group at week 1, week 2, and week 3 (82.5%, 80.0%, and 77.5%), respectively. Compared to a mean take rate of the NPWT group at week 1, week 2, and week 3 (83.0%, 71.8%, and 67.5%), respectively. Despite these differences, no statistical significant difference was found between the mean skin graft take rate of the NPWT and No-NPWT groups in the three weeks (Figure 3).

**Figure 4:**
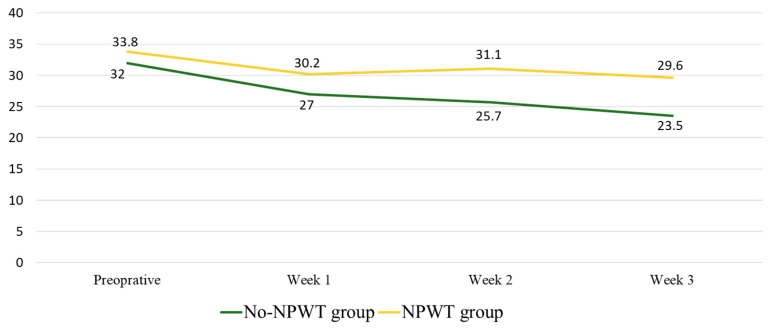
Total BWAT score mean compared between No-NPWT and NPWT groups.

Figure 4 shows the mean BWAT score for the patients before and after the treatment. The mean analysis of the BWAT score showed the following scores in the No-NPWT group: pre-operative, 32.0; week one, 27.0; week two, 25.7; and week three, 23.5. The NPWT group showed the following scores: pre-operative, 33.8; week one, 30.2; week two, 31.1; and week three, 29.6. These declining BWAT score trends were approximately similar in both groups, and there was no significant difference across all weeks (*p* = 0.74; *p* = 0.49; *p* = 0.21; *p* = 0.06, respectively).

Infection assessment comparison between the two groups showed one (12.5%) patient from the No-NPWT group had signs of inflammation and one (12.5%) patient had local infection in week three. On the other hand, the NPWT group had two (20%) patients with signs of inflammation and four (40%) patients with local infections. However, the difference between the two groups was not statistically significant (*p* > 0.05). 

The assessment of pain using the PPI scale was not statistically significant in either of the two groups at week one, week two, and week three (*p* = 0.59; *p* = 0.08; *p* = 0.35, respectively).

Hematoma formation was assessed in all patients after the treatment. Two patients in each group had hematoma with *p* = 0.86 at week one. At week two, the NPWT group had one patient with hematoma (*p* = 0.35), whereas there was no hematoma in either group at week three. The need to re-graft the same recipient area was not considered in the No-NPWT group. On the other hand, two (20%) patients in the NPWT group required re-grafting in the same recipient area. However, this difference was not statistically significant (*p* = 0.18).

## 4. Discussion

A split-thickness skin graft is one of the main tools used by the classical surgeon in the reconstruction of skin defects. There are three stages skin grafts must go through to ensure proper take rates: revascularization, lymphatic revascularization, and reinnervation [15]. The major reasons for skin graft loss are hematoma formation under the graft, infection of the grafted skin, and shear forces on the interface [16]. Despite extensive research that points to the benefit of NPWT in post-surgical wounds [17], as it promotes a moist wound-healing environment, reduces bacterial colony counts, increases granulation tissue formation, and removes edema, the clinical significance of NPWT over compressive bolster dressings (CBD) in skin grafts and the precise mechanism that provides wound healing have not been well established [17,18]. A recent systematic review of randomized controlled trials found no clear evidence of benefit for any use of NPWT. This was especially true when examining the role of NPWT as a postoperative dressing for STSG [17]. In this regard, our prospective cohort study aimed to evaluate wound and graft healing, infection rates, pain, and hematoma reduction after using NPWT compared to traditional conventional dressing. The current study showed no statistical difference in wound healing between the two groups. Additionally, no statistical difference in the take rate was found between the No-NPWT and NPWT groups in the three weeks. However, the mean take rate was higher in the second and third week favored the No-NPWT group, as a higher mean was found in the first week of the NPWT group compared to the No-NPWT, but lower means were found in the second and third weeks—83%, 71%, and 67% versus 82.5%, 80%, and 77%, respectively. No patient in the No-NPWT required re-grafting, but two patients with NPWT did.

One of the mechanisms by which NPWT is believed to promote the take rate of STSG is by inducing consistent and stable uniform pressure that immediately increases tissue perfusion [18]. The results of the present study suggest that using pressure of 125 or 75 mmHg in a clinical setting during the acute phase can improve take rate as was found in the first week; however, with time in the second and third weeks, the pressure effect may cause necrosis and decrease the volume of the skin substance, eventually reducing the take rate. This was also interesting for us as the VAC therapy left the skin site during the second and third week, but one of the possible interpretations is that VAC results in over-pressure to the attached skin which results in ongoing necrosis over time that can be then seen later in some areas of the transplanted skin. This is important for a surgeon who performs STSGs, as the later result will be always superior in terms of full take rate and the eventual scarring process in the future. The reported levels of negative pressure that must be applied to optimize tissue perfusion are in conflict within the literature. The current understanding implies that perfusion can be expected to be greatest at higher pressures. However, patients may experience pain when using high negative pressure [19], and there is a risk of developing ischemia in wounds [20]. Morykwas et al. reported that there was no positive effect on perfusion when a continuous suction of −125 mmHg was used [21]. Using a polarographic measuring technique, Lange et al. found that NPWT could significantly reduce tissue oxygenation in the foot [22]. In contrast, Timmers et al. found that perfusion was continuously enhanced as suction pressure was increased to a pressure of −500 mmHg or more [23]. The range of pressures that are widely used has been questioned, and factors such as tissue type, anatomical location, amount of edema present, and fibrosis may influence pressure range. Although the length of treatment depends on the treating physician’s goals of therapy, wound pathology, and wound size, there is still no clear guideline for how long the NPWT must be applied and when it should be removed; thus, further study is required to examine these factors. However, STSGs’ covering time should not exceed over five to seven days. As long-top dressing may provide an environment for growth of microorganisms and increase risk of infection [24], in this study, the first dressing was carried out at day five to seven after surgery and NPWT was removed from the NPWT group.

Another widely accepted mechanism among clinicians is that NPWT promotes wound healing through the continuous removal of interstitial fluid. Edema increases the pressure on wound tissue, which compromises the blood flow, then reduces nutrients and oxygen. These theories make intuitive sense; however, there are only a handful of studies that have directly measured this effect and examined the cytologic or molecular basis for the changes that NPWT presumably induces [21].

The present study has no striking significant statistical difference between the two groups in terms of patients’ demographics, co-morbidities, wound types, preoperative wound surface areas, and BWAT scores, except for wound sites, which was statistically significant (*p* = 0.04). The mean wound sizes of our two study groups were as follows: No-NPWT, 74.3%; and NPWT, 145%. Comorbidities such as congestive heart failure (CHF) have been shown to play a basic role in the impairment of healing processes resulting in graft failure; however, little literature has been published on the effects of CHF on skin graft take. Turissini et al. showed that patients with CHF had a 2.55-times higher risk for STSG failure because of tissue ischemia and edema [25]. The present study showed no statistically significant difference between two groups, although the presence of CHF was found to be the only factor independently predictive of failure to overall healing, delayed healing at 12 weeks, and the reduced rate of healing per week [26]. In terms of the effects of NPWT on infection, previous studies have produced mixed results. The authors of a meta-analysis stated that compared to standard surgical dressings, NPWT significantly reduced the rate of wound infections and seroma development in closed surgical wounds [12]. An article published in 2017 explained that NPWT results in decreased bacterial load due to the effect of suction, increased angiogenesis, and blood perfusion leading to an increase in the inflow of oxygen in the wound tissue, resulting in increased infection resistance [27], while another study conducted in 2018 showed no significant impact of NPWT on wound infection and bacterial colonization [10,13]. Although it is consistent with what has been found in this study as there was no significant difference between the NPWT group and No-NPWT group in infection assessment comparison (*p* > 0.05), we assume that the risk of local infection with the use of NPWT is higher as one (12.5%) patient from the No-NPWT group had signs of inflammation and one (12.5%) patient had local infection in week three. On the other hand, the NPWT group had two (20%) patients with signs of inflammation and four (40%) patients with local infections.

One of the most common side effects of NPWT is pain, which impacts patients’ quality of life which leads patients to ask to cease the therapy [28]. Several studies recorded levels of pain in patients applying NPWT. Levels of pain differ with the type of filler and dressing used in the treatment [29,30]. Most patients feel pain during dressing changes due to the torn-away granulation tissue that has grown in the micropores of the NPWT foam. To decrease such pain, using gauze and infiltrating the wound filler with saline or local lidocaine before dressing changes have been suggested [31]. Demography, comorbidity, parameters of the wound, wound bed preparation, and the dressing used after STSG procedures are all factors that might affect the final take rate. However, the most common cause of the failure of skin grafts is the formation of hematoma, as it affects proper graft attachment and revascularization [16]. The role of NPWT is to improve the take rate before STSG by improving wound bed preparation and after STSG by decreasing the rate of hematoma formation [32]. This study does have some limitations. Although baseline characteristics of the two groups were similar among those who completed the follow-up, the bias of the small sample could be an issue in this study. As well as this, our study has a lack of blinding; thus, the generalizability may be limited. However, using large-scale randomized controlled clinical trials would minimize bias and maximize the validity of the results.

## 5. Conclusions

This study aimed to compare the outcomes of the application of NPWT versus conventional dressing on STSGs after soft-tissue reconstruction. Our study showed no statistically significant difference in terms of the skin graft take rate between the two groups. This contribution will lead to reduced cost and increased revenue by reducing the unnecessary use of equipment with intended outcomes for patients undergoing reconstruction of skin defects with STSG. We believe that our study may contribute to enhance the scientific literature in STSG dressing research including negative pressure therapy. There were no significant differences in demographic characteristics, baseline wound characteristics, or healing. Although there were differences in the infection, hematoma, and the need for re-grafting the same recipient area rates between the two groups, they were not statistically significant. Some limitations to this study should be noted. The bias of the small sample could be an issue. Therefore, this appears to be a potentially fruitful avenue for future studies with a larger sample size, and would minimize bias, maximize the validity of the results, and enhance the scientific literature in STSGs dressing research including negative-pressure therapy. 

## Figures and Tables

**Table 1:** Baseline patients’ characteristics compared between No-NPWT and NPWT groups.

Variables		NPWT Application		Total	*p*-Value
		No, (*n* = 8)	Yes, (*n* = 10)		
Age, mean (SD) years		43.8 (11.9)	52.6 (19.7)		0.289
BMI, mean (SD)		29.9 (6.9)	31.1 (7.0)		0.725
Nationality, *n* (%)	Saudi	7 (87.5%)	9 (90.0%)	16 (88.8%)	0.867
	Non-Saudi	1 (5.5%)	1 (10.0%)	2 (11.1%)	
Gender, *n* (%)	Male	7 (58.3%)	5 (50.0%)	12 (66.7%)	0.094
	Female	1 (5.5%)	5 (50.0%)	6 (33.3%)	
Area of residency, *n* (%)	Makkah	2 (25.0%)	7 (70.0%)	9 (50.0%)	0.139
	Jeddah	1 (5.5%)	0	1 (5.5%)	
	Madinah	0	1 (10.0%)	1 (5.5%)	
	Taif	2 (25.0%)	0	2 (11.1%)	
	Abha	2 (25.0%)	0	2 (11.1%)	
	Jazan	1 (5.5%)	1 (10.0%)	2 (11.1%)	
	Other	0	1 (10.0%)	1 (5.5%)	
Co-morbidities, *n* (%)	Heart Disease	1 (5.5%)	4 (40.0%)	5 (27.8%)	0.196
	Diabetes Mellitus	0	2 (20.0%)	2 (11.1%)	0.180
	Hypertension	0	2 (20.0%)	2 (11.1%)	0.180
	Asthma	1 (5.5%)	1 (10.0%)	2 (11.1%)	0.867
	Anemia	2 (25.0%)	0	2 (11.1%)	0.094
Lifestyle, *n* (%)	Smoking	1 (5.5%)	0	1 (5.5%)	0.250
Medication, *n* (%)	Aspirin	5 (62.5%)	2 (20.0%)	7 (38.9%)	0.066
	Warfarin	2 (25.0%)	1 (10.0%)	3 (16.7)	0.396
Type of wound, *n* (%)	Acute	5 (62.5%)	4 (40.0%)	9 (50.0%)	0.343
	Chronic	3 (37.5%)	6 (60.0%)	9 (50.0%)	
Site of wound, *n* (%)	Lower limp	2 (25.0%)	8 (80.0%)	10 (55.6%)	0.043
	Upper limp	5 (62.5%)	1 (10.0%)	6 (33.4%)	
	Thorax	0	1 (10.0%)	1 (5.5%)	
	Abdomen	1 (5.5%)	0	1 (5.5%)	
Preoperative wound surface area cm^2^, mean (SD)		74.3 (59.5)	145.9 (164.3)		0.226
Preoperative BWAT score, mean (SD)		32.0 (11.5)	33.8 (11.3)		0.744

## Data Availability

All data generated or analyzed during this study are included in this published article.
